# Supplementary material for: Multiscale interface engineering enables strong and water resistant wood bonding
Source: Nat Commun. 2025 Aug 25;16:7902. doi: 10.1038/s41467-025-63239-y (PMC12378965; doi:10.1038/s41467-025-63239-y)
Supplement: Supplementary file 1 — Supplementary Information [file 41467_2025_63239_MOESM1_ESM.pdf]

Supplementary Information for

Multiscale interface engineering enables strong and water resistant wood bonding

Shiying Zhang[1], Salla Koskela[1,2], Halvar Meinhard[1], Muhammad Awais[1,3], Paavo Penttilä[1], Markus B. Linder[1,2], Shennan Wang[1]*, and Lauri Rautkari[1]*

[1]Department of Bioproducts and Biosystems, School of Chemical Engineering, Aalto University; Espoo, FI-02150, Finland

[2]Center of Excellence in Life-Inspired Hybrid Materials, Aalto University, Espoo, 02150, Finland

[3]Faculty of Science and Technology, Norwegian University of Life Sciences, Pb 5003, Ås, 1433, Norway

*Corresponding author, e-mail:

Lauri Rautkari, lauri.rautkari@aalto.fi;

Shennan Wang, shennan.wang@aalto.fi

This Supplementary Information includes:

Supplementary Notes 1-4

Supplementary Fig. 1-26

Supplementary Table 1-3

Supplementary References

Supplementary Note 1: Bonding test with Norway spruce

The Norway spruce (*Picea Abies*) specimens were successfully bonded with 5 wt.% pulp-IL, and the bonding strength is presented in Supplementary Fig. 11 below. As expected, the bonding strength of spruce (14.0 ± 0.9 MPa) was lower than that of Pine (19.6 ± 1.3 MPa), which can be attributed to the lower density (320 kg m^{-3}) as well as the lack of large, window-like, cross-field pits. Nevertheless, the shear strength measured for spruce bonded with pulp-IL remains higher than that of solid spruce (5-10 MPa).[1] Additionally, fluorescence microscopy (Supplementary Fig. 12) revealed that the cell lumina at the bonding interface in spruce were filled with regenerated cellulose, demonstrating a similar microstructure to that observed in pine wood.

Supplementary Note 2: Effect of pulp concentration on the bonding performance

The flow behavior of pulp-IL solution at difference pulp concentrations is shown in Supplementary Fig. 16. At 20 °C, the steady state viscosities of 3, 5, and 8 wt.% pulp-IL solutions differed by approximately one order of magnitude. However, at 140 °C, the viscosities dropped significantly, and the difference between the 5 and 8 wt.% solutions became negligible. The difference in viscosity is expected to influence the penetration of pulp-IL solutions into wood. As shown in the fluorescence microscopy images (Supplementary Fig. 18), the wood bonded with 3 wt.% pulp-IL solution had a thicker bonding interface with regenerated cellulose primarily detectable at the lumina surface. This suggests better penetrability into the wood structure due to the much lower viscosity but a reduced capacity for network formation due to the lower cellulose concentration. In contrast, wood bonded with 5 wt.% and 8 wt.% pulp-IL solutions showed thinner bonding interface and fully filled lumina. The lumen filling behavior of 8 wt.% pulp-IL closely resembled that of 5 wt.% pulp-IL. Due to differences in lumen filling, wood bonded with 3 wt.% pulp-IL showed lower shear strength of 15.9 ± 1.1 MPa (Supplementary Fig. 19), while 8 wt.% pulp-IL bonded wood reached 20.3 ± 0.5 MPa, similar to the result obtained with 5 wt.% pulp-IL. In conclusion, pulp concentration lower than 5 wt.% may impair wood bonding performance due to limited network formation in the lumina at the bonding interface. A pulp concentration higher than or equal to 5 wt.% is required to achieve the best bonding performance.

Supplementary Note 3: Effect of various parameters on the bonding performance of pulp-IL bonded wood

Effect of regeneration methods

To investigate the kinetics of cellulose regeneration induced by water, we performed regeneration process by using two alternative methods: 1) rapid regeneration by exposure to water steam (50 mL per sample) with an iron (Supplementary Movie 3), and 2) slow regeneration in a 65% RH environment for one month (Supplementary Fig. 20). Both methods resulted in lower shear strength (11.6 ± 1.2 MPa and 13.2 ± 0.7 MPa, respectively) compared to regeneration with water rinsing for overnight (19.6 ± 1.3 MPa). However, the bonding strengths achieved with both methods were still comparable to that of solid wood. These findings indicate that the effectiveness of the regeneration process significantly influences the overall bonding performance.

Effect of hot-pressing temperature

We bonded wood using 5 wt.% pulp-IL solution at both room temperature (20 °C) and 90 °C, right above the dissolution temperature (85 °C) for cellulose (Supplementary Fig. 21), both at a pressure of 1.5 MPa. Notably, the wood samples bonded at 20 °C exhibited delamination during water regeneration, indicating insufficient interfacial adhesion at low temperature. The wood samples bonded at 90 °C demonstrated a reduced bonding strength of 11.5 MPa compared to 19.6 ± 1.3 MPa of those bonded at 140 °C and 1.5 MPa. These results suggest that effective wood bonding requires sufficient heat to promote cellulose penetration and to activate the softening effect of the IL on cell wall components at the bonding interface.

Effect of hot-pressing duration

As shown in Supplementary Fig. 22, wood assemblies subjected to 10 min hot-pressing achieved a bonding strength of 17.5 ± 1.2 MPa, while those pressed for 60 min yielded 19.1 ± 0.6 MPa. The 10 mins hot pressed samples show slightly lower strength compared to those pressed for 30 min (19.6 ± 1.3 MPa), whereas the 60 min hot-pressing results were comparable to the 30 min samples. These observations indicate that extending the hot-pressing duration up to 30 min can enhance bonding performance, likely due to improved penetration of cellulose solution into the cell lumina and formation of a more integrated bonding network. From an

industrial perspective, however, a 10 min hot-pressing duration is sufficient to produce bonded wood with good bonding strength, balancing performance with process efficiency.

Effect of hot-pressing pressure

As shown in Supplementary Fig. 23, wood bonding was performed using a 5 wt.% pulp-IL solution at 140 °C under reduced hot-pressing pressure of 0.1 and 1 MPa. Notably, wood samples bonded under 0.1 MPa pressure exhibited complete delamination after water regeneration, indicating poor interfacial adhesion. In contrast, samples bonded under 1 MPa pressure did not show delamination but they demonstrated reduced bonding strengths of 14.3 ± 0.7 MPa compared to those bonded at 1.5 MPa (19.6 ± 1.3 MPa). These results suggest that effective wood bonding requires sufficient pressure to promote cellulose penetration and activate the softening effect of ILs on cell wall components at the bonding interface.

Effect of knots in the wood adherends

Regarding the implementation of less ideal wood substrates in industrial applications, wood specimens containing knots were bonded using a 5 wt.% pulp-IL solution (Supplementary Fig. 24). The corresponding shear strength was evaluated, and both the measured values and the image of fractured samples are demonstrated in Supplementary Fig. 24b. As shown in Supplementary Fig. 24a, the wood substrates containing knots were successfully bonded; notably, no visible delamination occurred even after water washing. The shear strength of the samples containing knots (9.8 ± 2.6 MPa) was lower than that of the defect-free samples (19.6 ± 1.3 MPa), likely due to the structural discontinuities introduced by the knots. Nevertheless, these results suggest that while knots may reduce the overall shear strength, they do not inhabit the bonding capability of the proposed method. Moreover, the shear strength remains within a range that is comparable to typical values for the substrates, indicating the potential suitability of this bonding approach for industrial applications involving less ideal wood materials.

Supplementary Note 4: Purification, recovery, and re-use of IL [emim][OAc]

To evaluate the reusability of [emim][OAc] in our process, we recovered [emim][OAc] from the wastewater collected during the water regeneration (Supplementary Fig. 25c). The wastewater containing mainly [emim][OAc] and water was first vacuum filtrated through a membrane filter (Polycarbonate, pore size: 0.1 μm) to remove insoluble fractions

(Supplementary Fig. 25a). The purified [emim][OAc]/water mixture was then concentrated via rotary evaporation (Supplementary Fig. 25b). To further eliminate residual water, the concentrated solution was then oven-dried at 105 °C overnight. The recovered [emim][OAc] demonstrates a color change from yellowish to brownish; however, no noticeable changes in its chemical characteristics were detected by FTIR (Supplementary Fig. 25d), except for slight increase in -OH band intensity at high wavenumber, possibly due to difficult-to-remove residue water.

Subsequently, 5 wt.% pulp was dissolved using this recovered [emim][OAc] to prepare a cellulose-IL solution. Wood substrates were bonded using this solution (Supplementary Fig. 25e), and successfully bonding was achieved. The bonded wood samples exhibited a shear strength of 13.7 MPa (Supplementary Fig. 26), demonstrating the effective reusability of the recovered [emim][OAc]. The reduced shear strength might be due to reduced cellulose solubility in the presence of aging products of [emim][OAc], such as *N*-methylimidazole and imidazole, which share similar structure as [emim][OAc] and thus can hardly be identified by FTIR.[2]

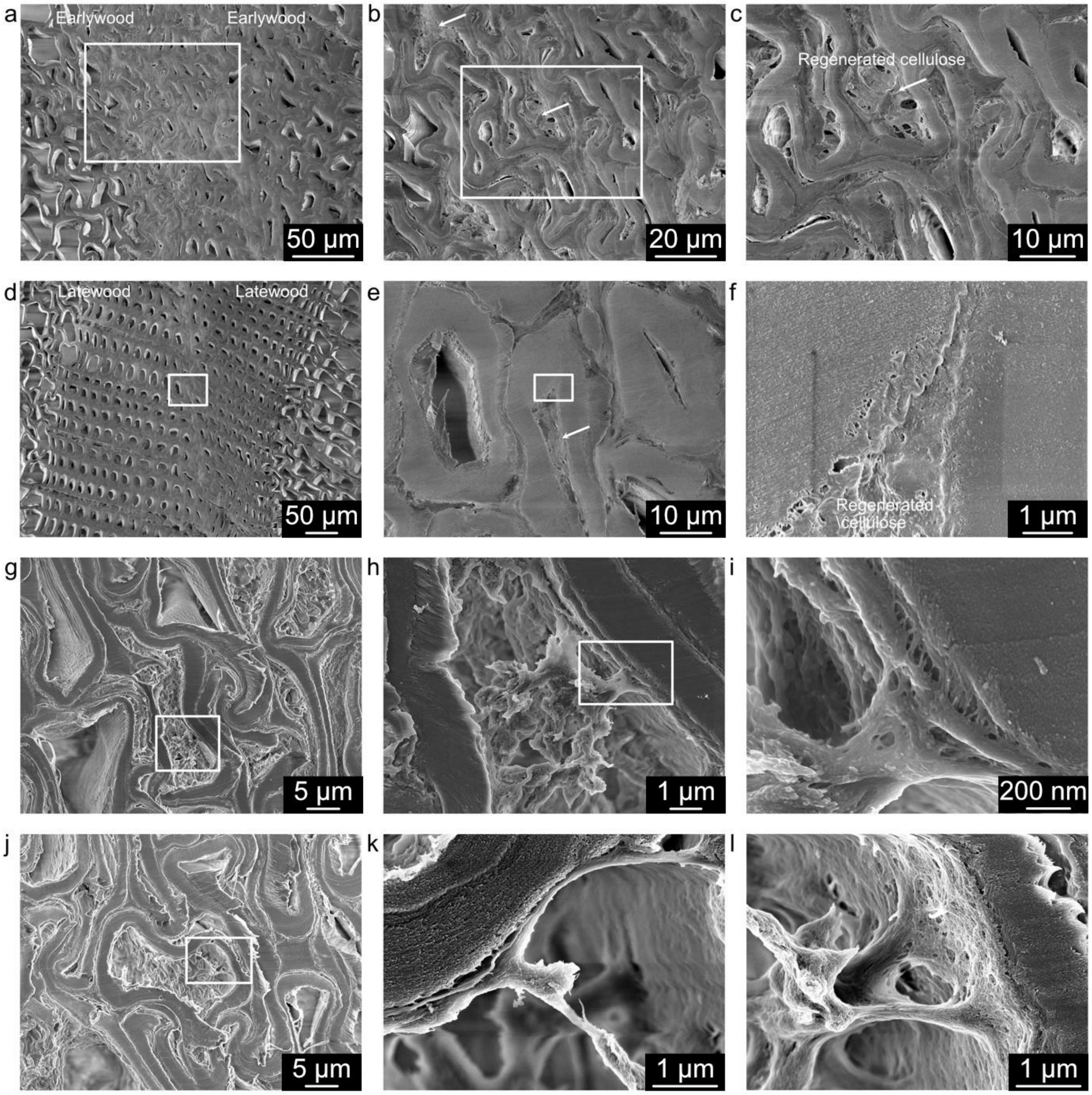

Supplementary Fig. 1. SEM images illustrating the morphology of the cell wall and lumen at the bonding interface. The cell walls are densely interlocked and the lumina are filled with regenerated cellulose. a) The bonding line morphology at the junction of earlywood-earlywood, along with its magnified regions b) and c). d) The bonding line morphology at the junction of latewood-latewood, along with its magnified region e) and f). g-i) and j-e) Close-up images demonstrating the morphology of regenerated cellulose network in a cell lumen, where the network of regenerated cellulose is shown as strongly anchored to the lumen surface.

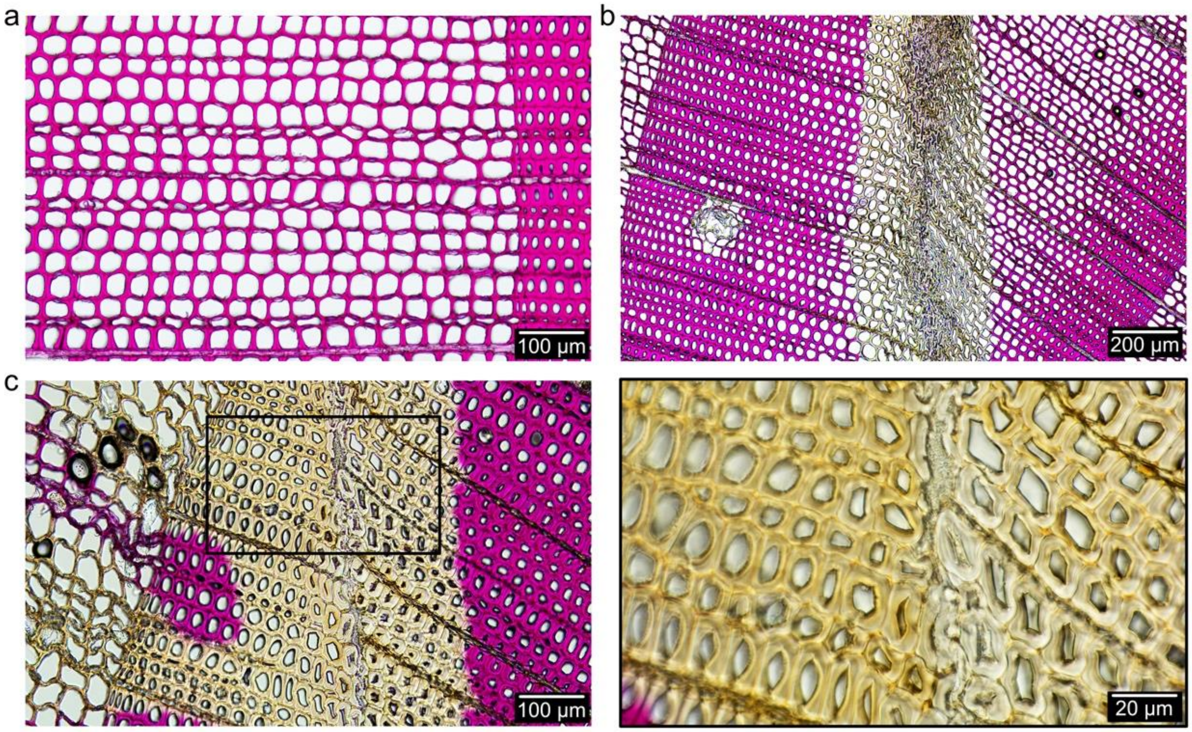

Supplementary Fig. 2. Light microscopy images of wood samples subjected to Wiesner staining. Images of a) transverse section of Scots pine control sample, b) bonding interface morphology of earlywood-earlywood shows the appearance of post-Wiesner staining, c) latewood-latewood bonding interface morphology with Wiesner staining appearance, along with a magnified section image on the right.

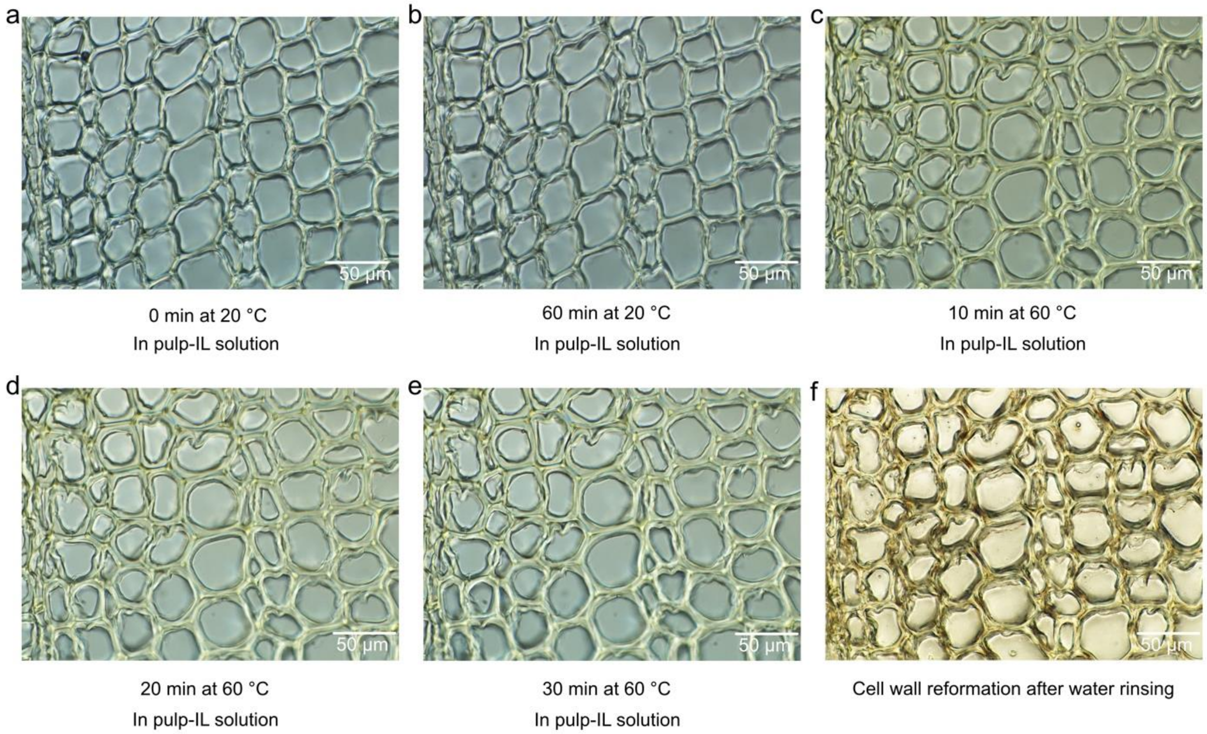

Supplementary Fig. 3. Light microscopy images demonstrating the dynamic process of wood cell wall swelling in the pulp-IL solution at 60 °C and reformation upon water washing. a) Wood cell wall morphology in the pulp-IL solution in the initial state at 20 °C. b) Wood cell wall morphology after emerging in pulp-IL solution for 60 min at 20 °C. c) Wood cell wall swelling in pulp-IL solution after 10 min heating at 60 °C. d) Wood cell wall swelling in pulp-IL solution after 20 min heating at 60 °C. e) Wood cell wall swelling in pulp-IL solution after 30 min heating at 60 °C. f) Wood cell wall reformation after water rinsing.

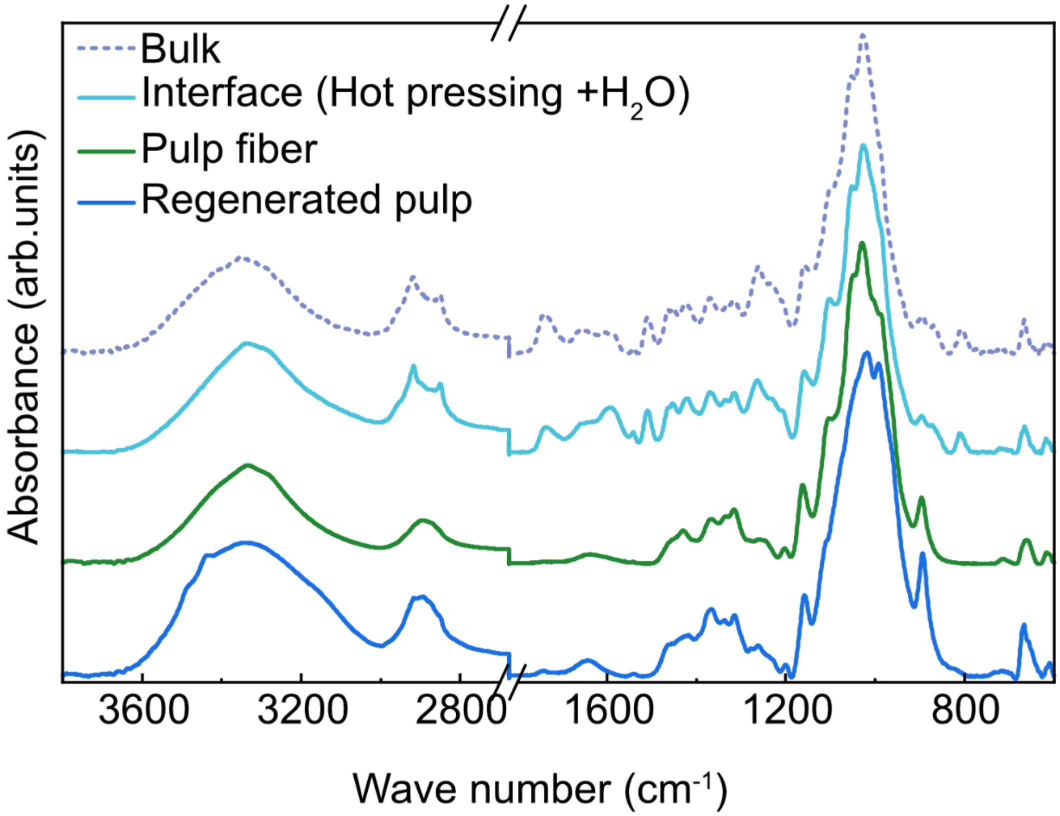

Supplementary Fig. 4. The Attenuated Total Reflectance-Fourier Transform Infrared (ATR-FTIR) spectra of bonded wood and regenerated pulp film prepared on glass slide.

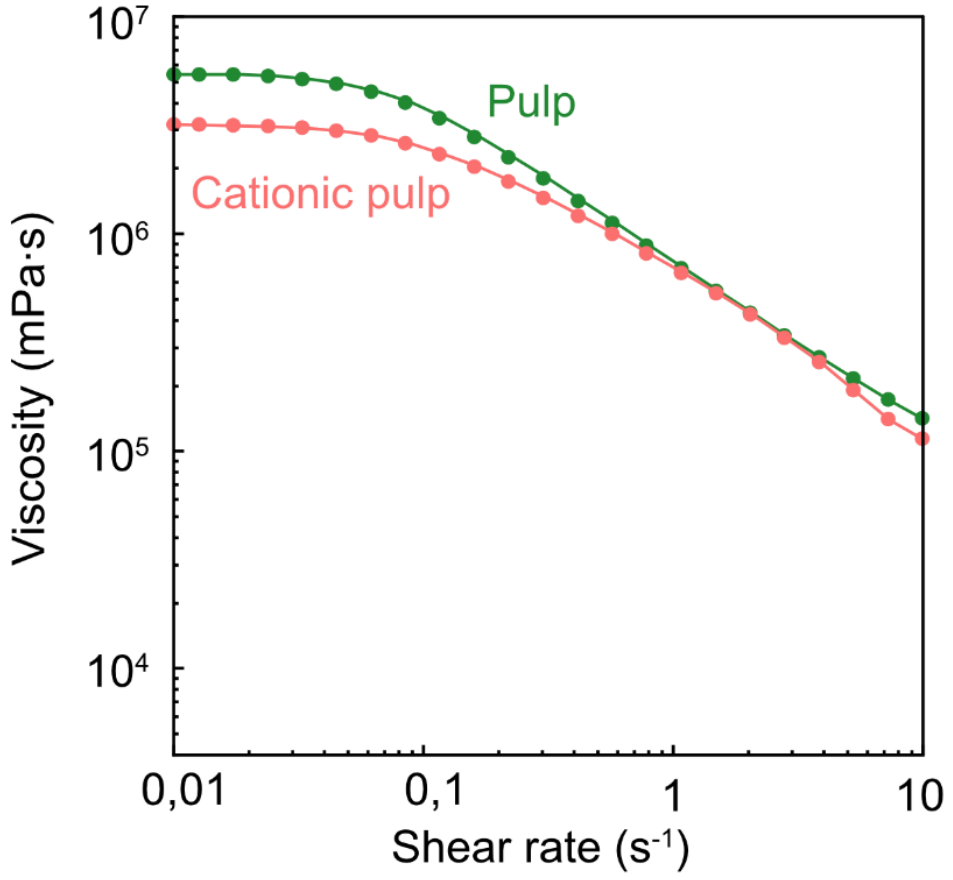

Supplementary Fig. 5. Flow curves of original pulp solution and cationic pulp solution at room temperature.

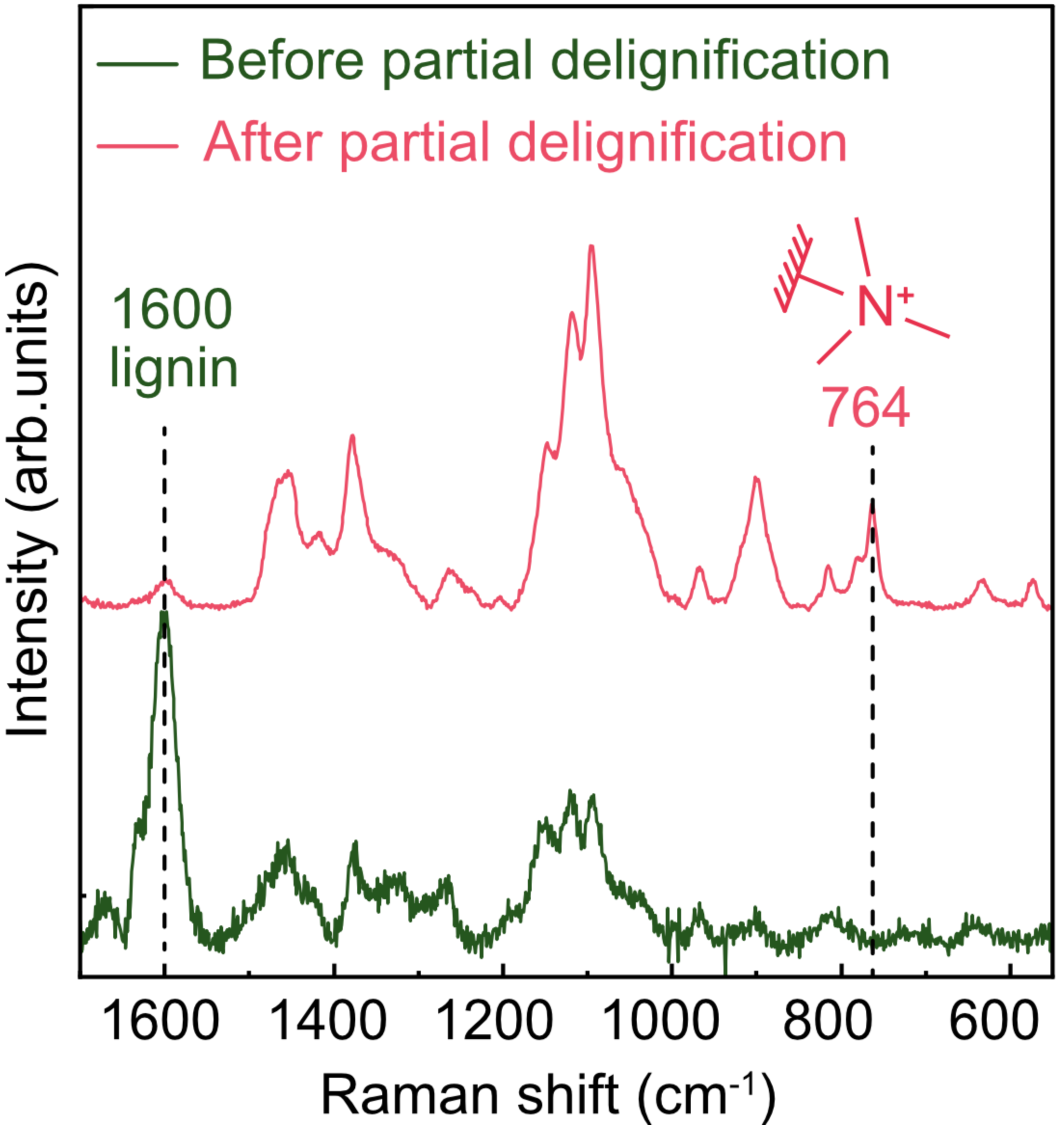

Supplementary Fig. 6. Raman spectra of the cell wall at the bonding interface before and after partial delignification.

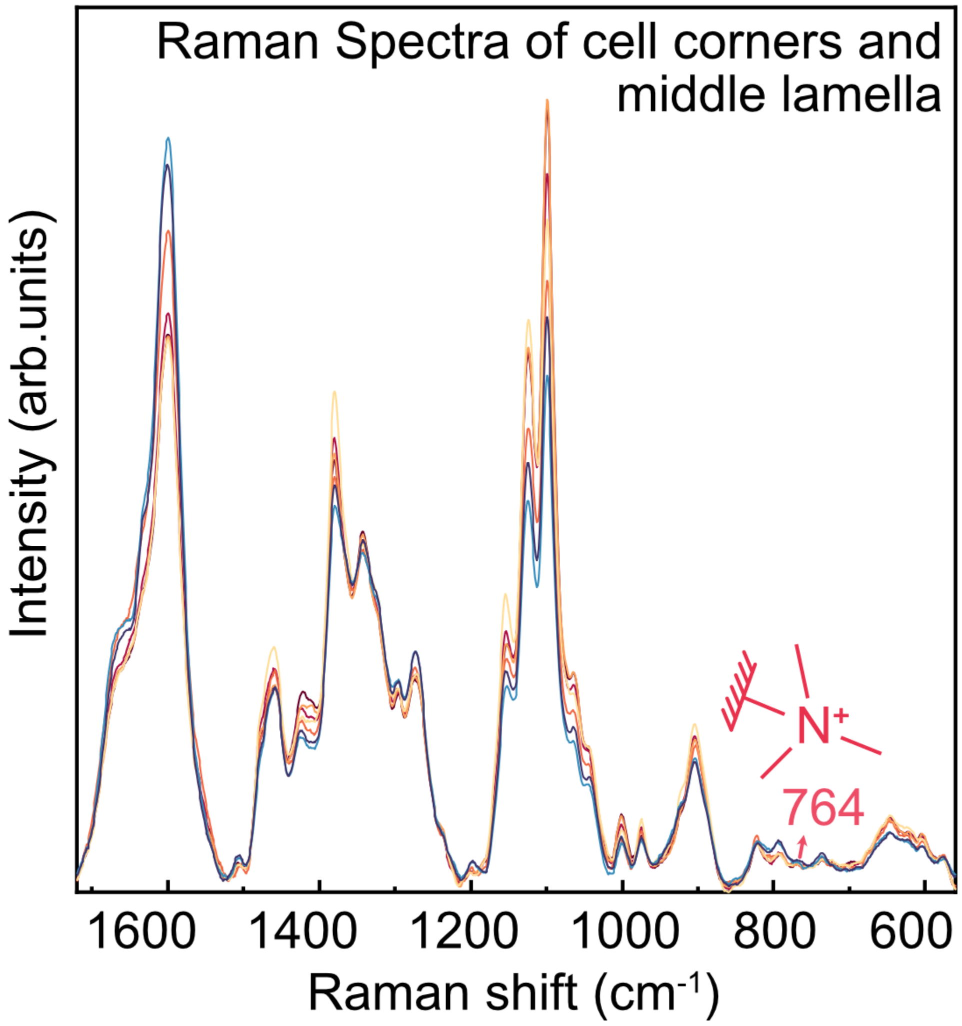

Supplementary Fig. 7. Raman spectra extracted from cell corners and middle lamella at the bonding interface.

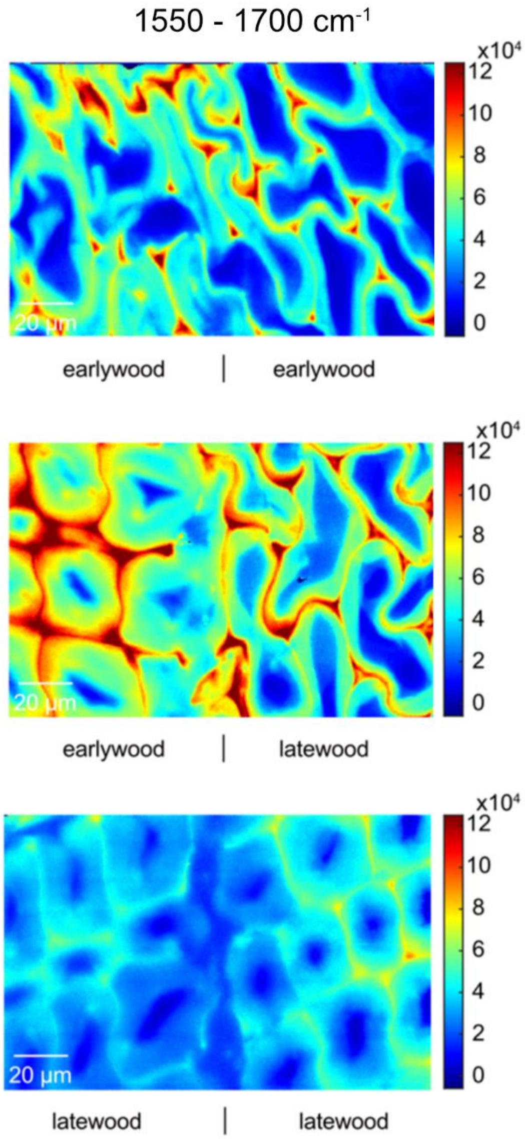

Supplementary Fig. 8. Raman mapping on the transverse section of bonding interface showing the distribution of lignin between different types of wood tracheids. The colored map demonstrates the integration of Raman bands from 1550-1700 cm^{-1}, corresponding to the peak area of 1600 cm^{-1}.

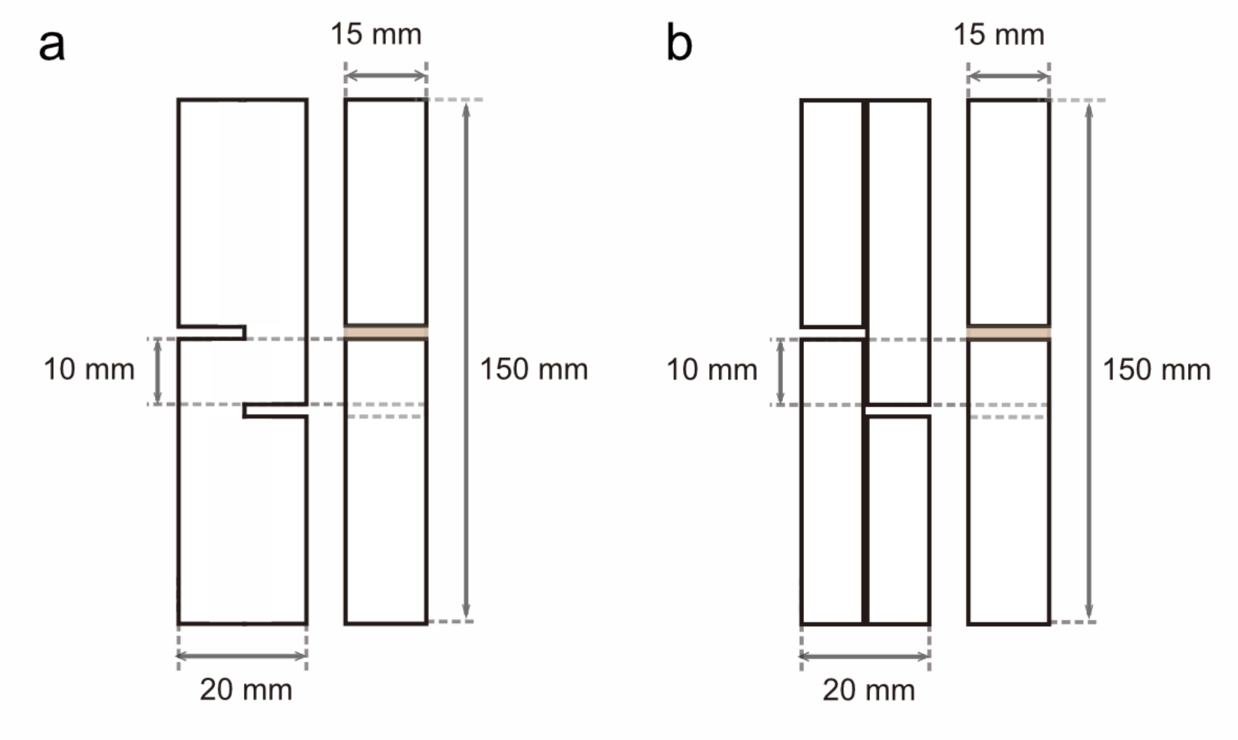

Supplementary Fig. 9. The geometries of specimens for tensile shear test. a) for solid pine wood (control), b) for bonded wood.

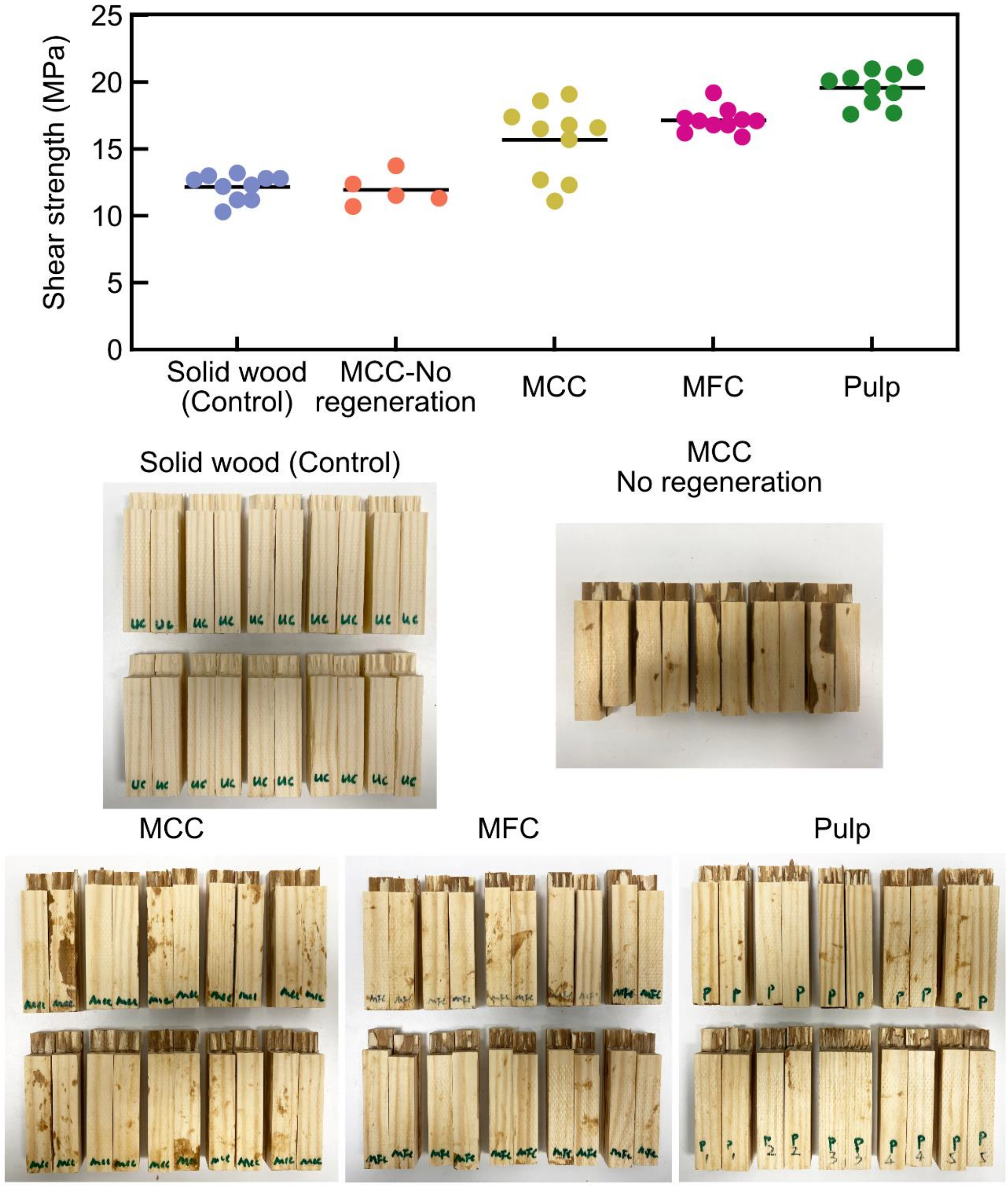

Supplementary Fig. 10. Shear strength of wood bonded with different cellulose-IL solutions and corresponding photos of fractured samples. Horizontal lines indicate mean value.

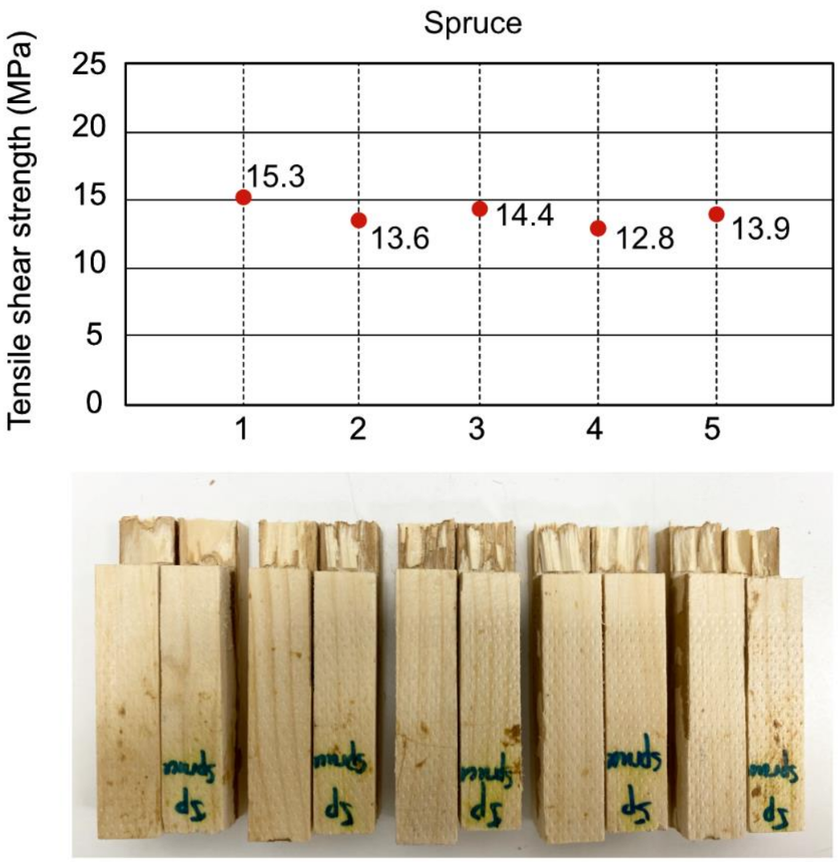

Supplementary Fig. 11. The shear strength and the photo of fractured spruce samples bonded with 5 wt.% pulp-IL solution.

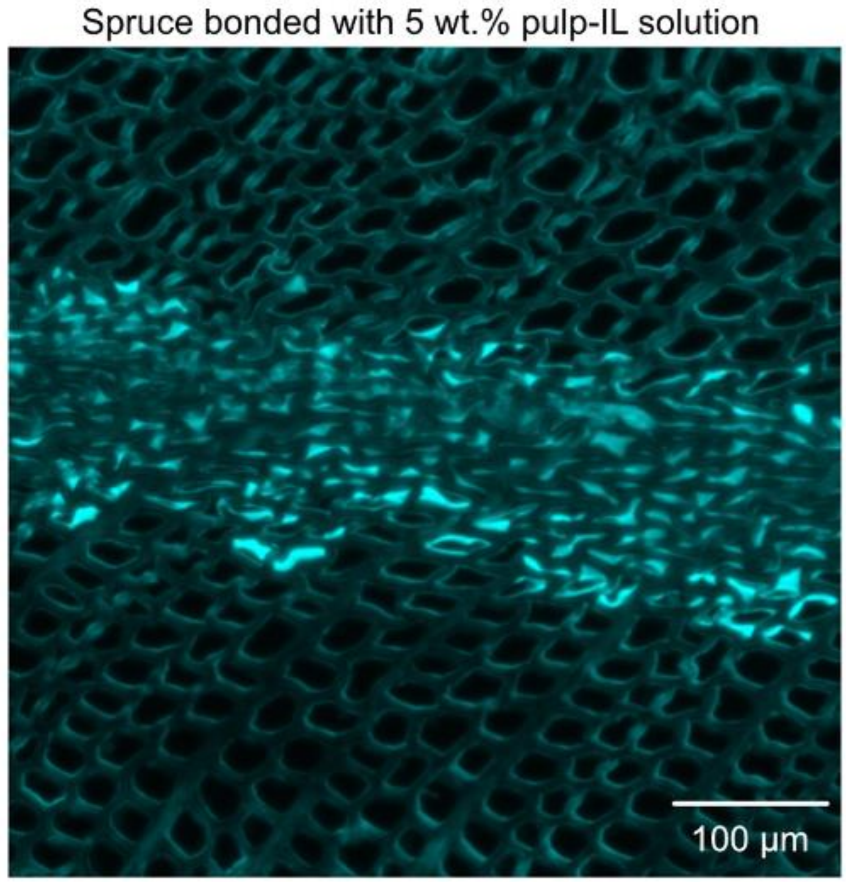

Supplementary Fig. 12. Fluorescence microscopy images showing calcofluor white stained cross-sections of wood bonded using 5 wt.% pulp-IL solution.

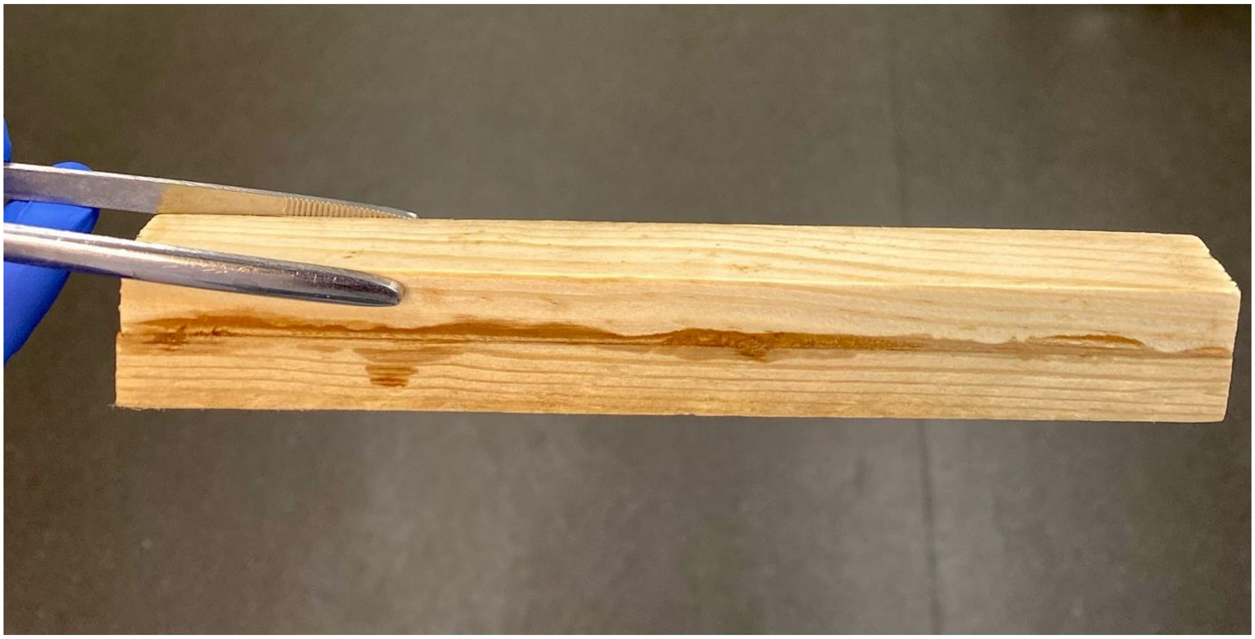

Supplementary Fig. 13. Pulp-bonded wood subjected to delamination test. Bonded wood was boiled at 100 °C for 6 h and followed by 2 h of water soaking at 20 °C.

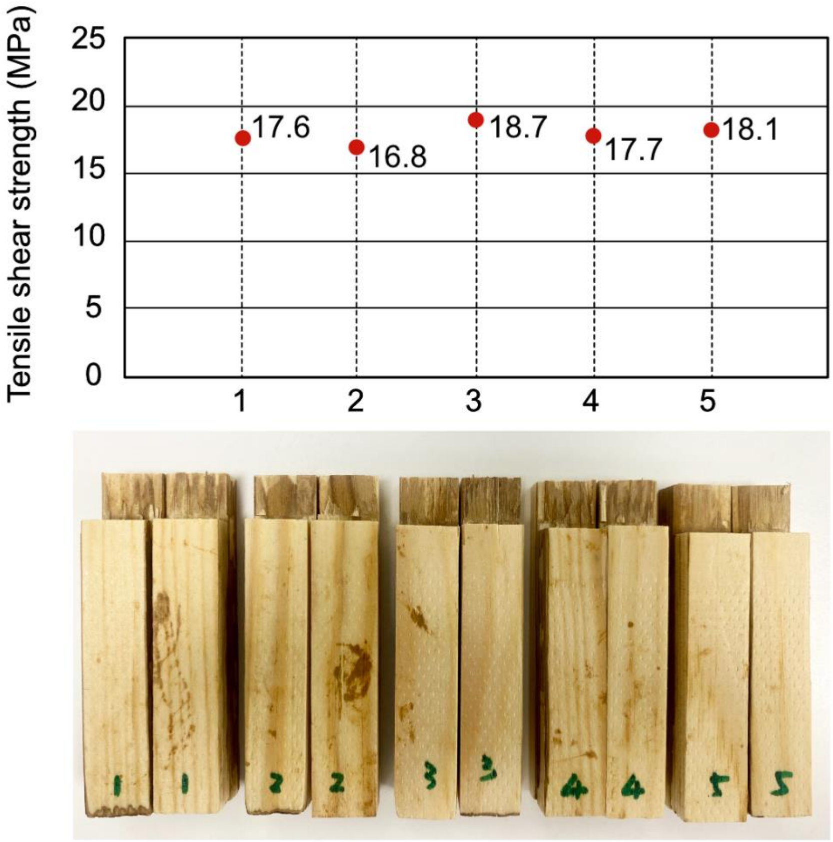

Supplementary Fig. 14. Shear test result of pulp-bonded wood after delamination test. Bonded wood was boiled at 100 °C for 6 h and followed by 2 h of water soaking at 20 °C.

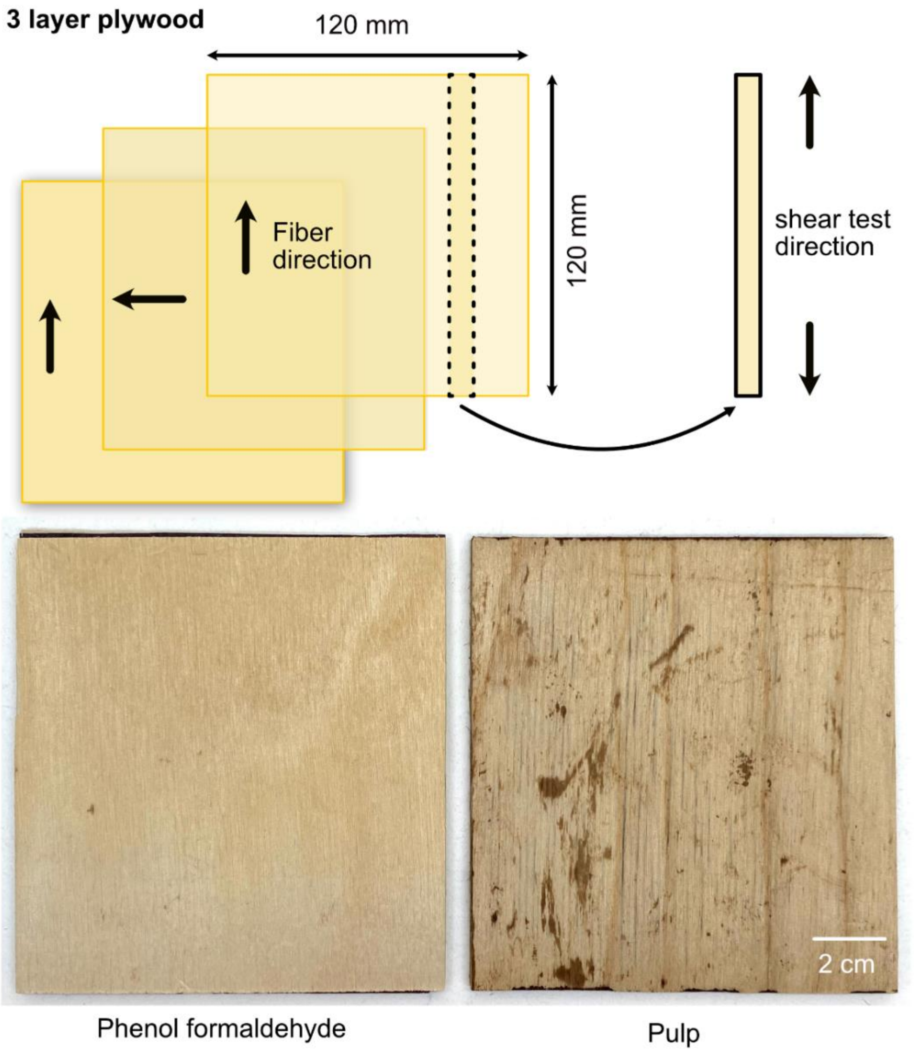

Supplementary Fig. 15. The 3-layer plywood bonded by Pulp-IL solution and commercial phenol formaldehyde adhesive.

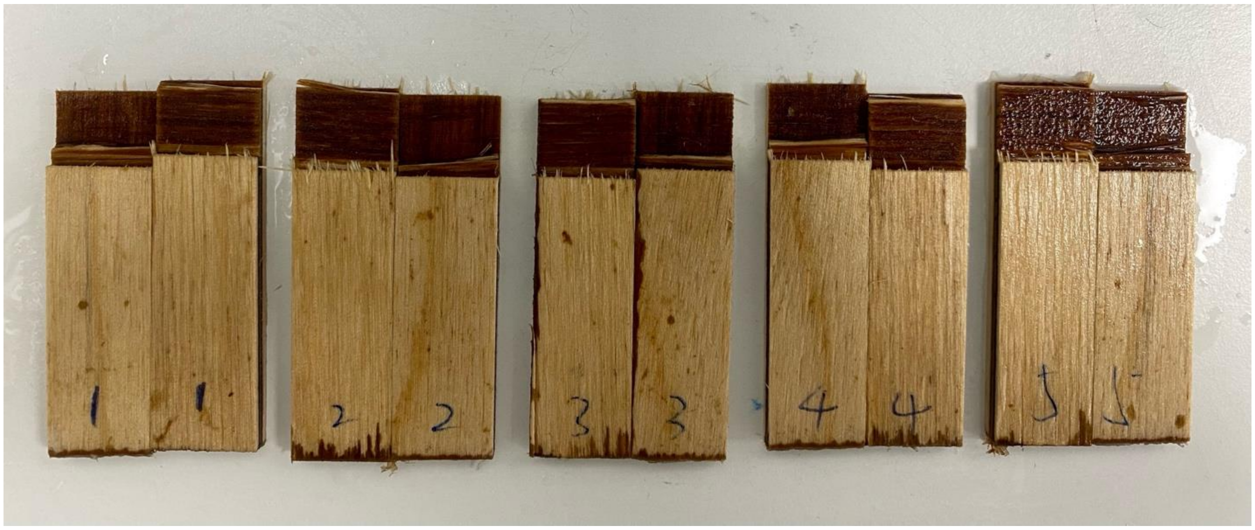

Supplementary Fig. 16. The 3-layer plywood bonded by Pulp-IL solution after the wet bonding strength test.

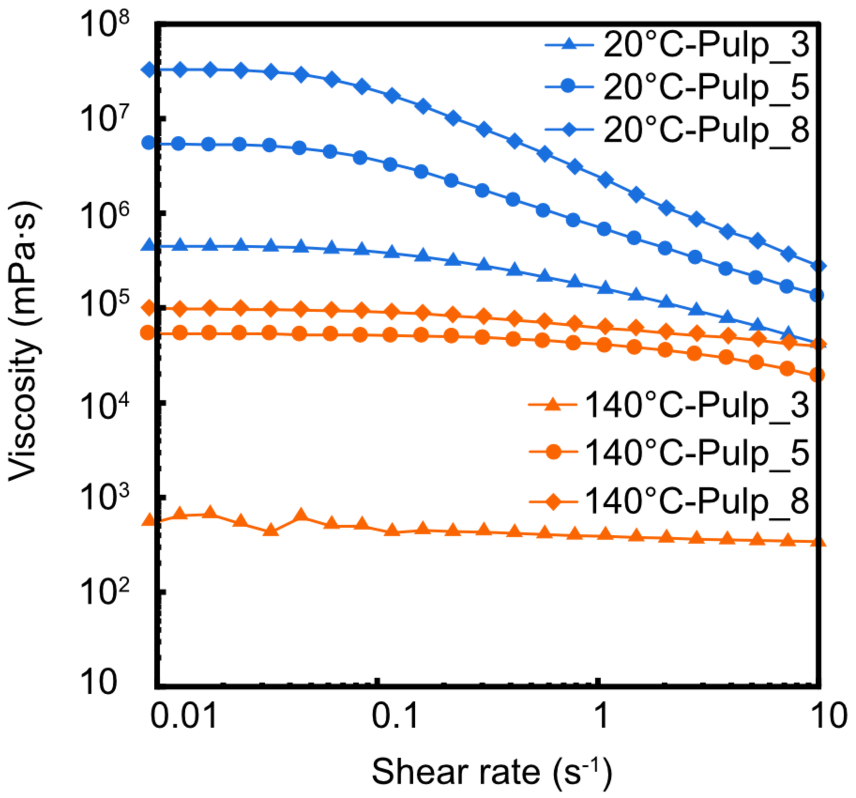

Supplementary Fig. 17. Flow curves of pulp-IL solution containing 3 wt.%, 5wt.% and 8 wt.% pulp at 20 °C and 140 °C.

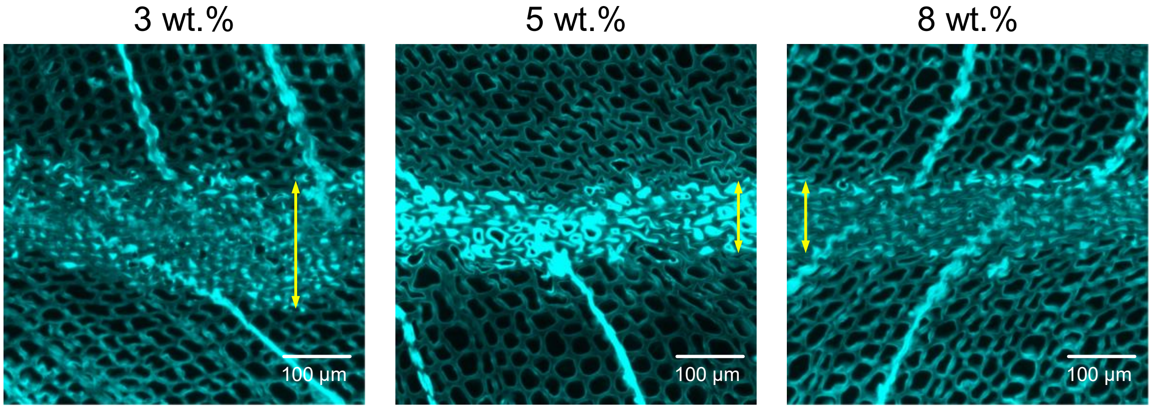

Supplementary Fig. 18. Fluorescence microscopy images showing calcofluor white stained cross-sections of wood bonded using 3 wt.% pulp-IL solution and 8 wt.% pulp-IL solution.

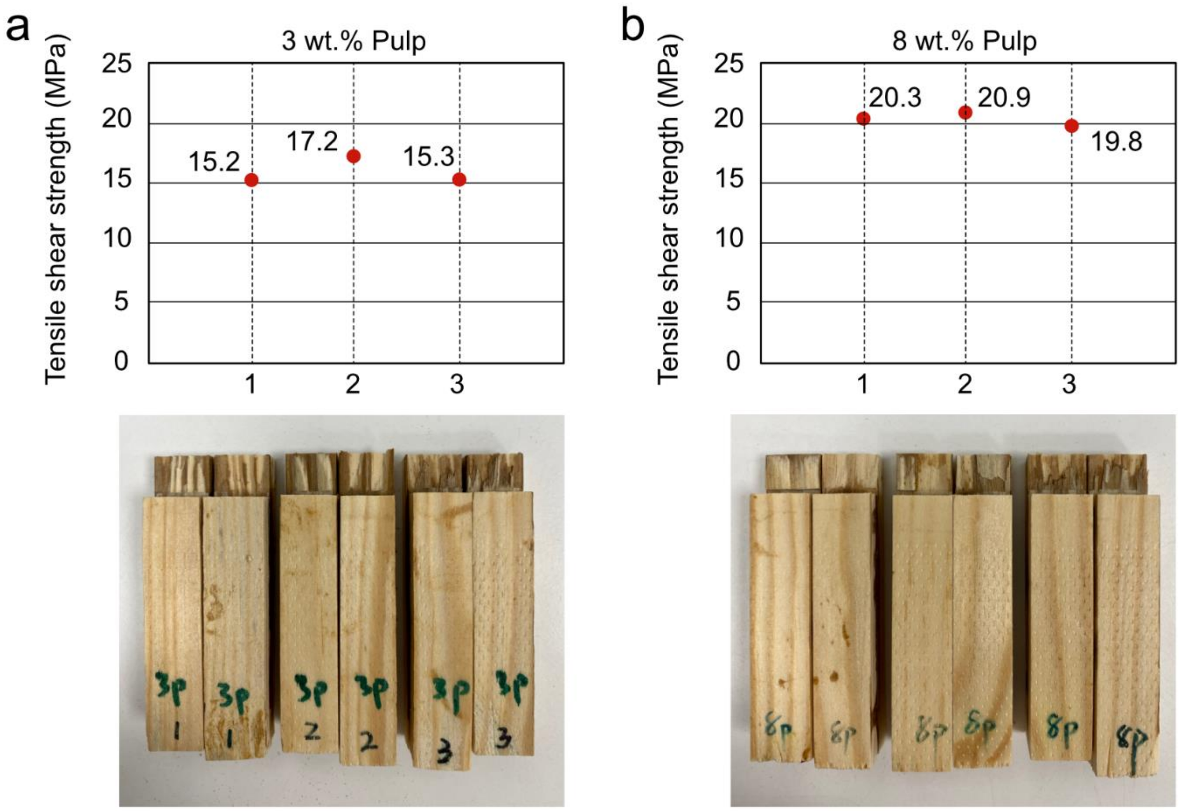

Supplementary Fig. 19. The summary of shear strength and the photos of fractured wood samples bonded with different concentrations of pulp-IL solution and hot-pressed for 30 min at 140 °C with 1.5 MPa pressure. a) wood sample bonded with 3 wt.% pulp-IL solution. b) wood sample bonded with 8 wt.% pulp-IL solution.

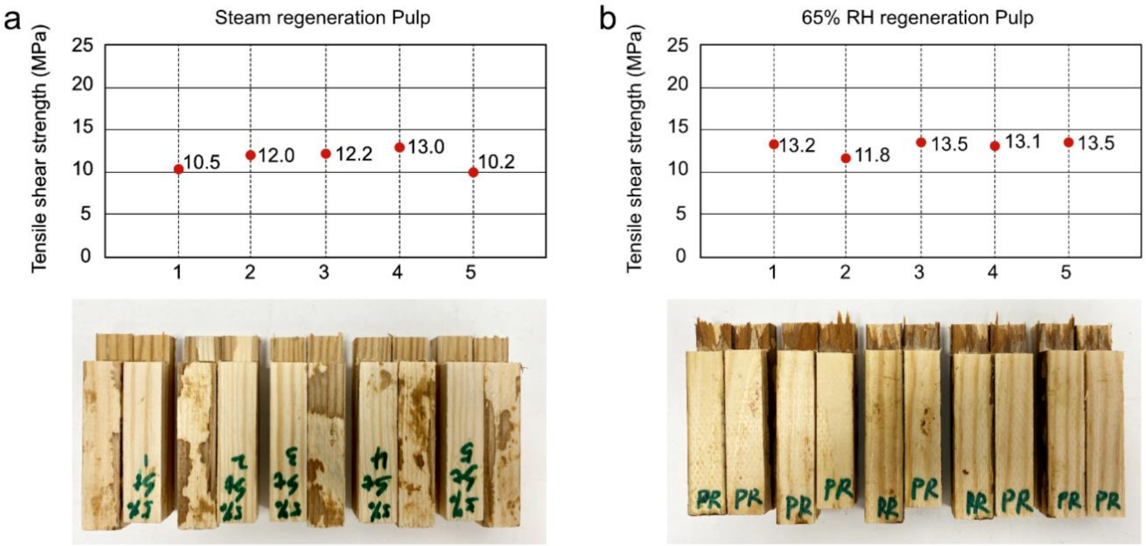

Supplementary Fig. 20. Effect of regeneration methods. The bonding strengths and the photos of fractured wood samples bonded with 5 wt.% pulp-IL solution hot-pressed for 30 min at 140 °C with 1.5 MPa pressure, following different regeneration methods. a) Steam regeneration of bonded wood sample. b) Bonded wood sample regenerated in a 65 % RH room.

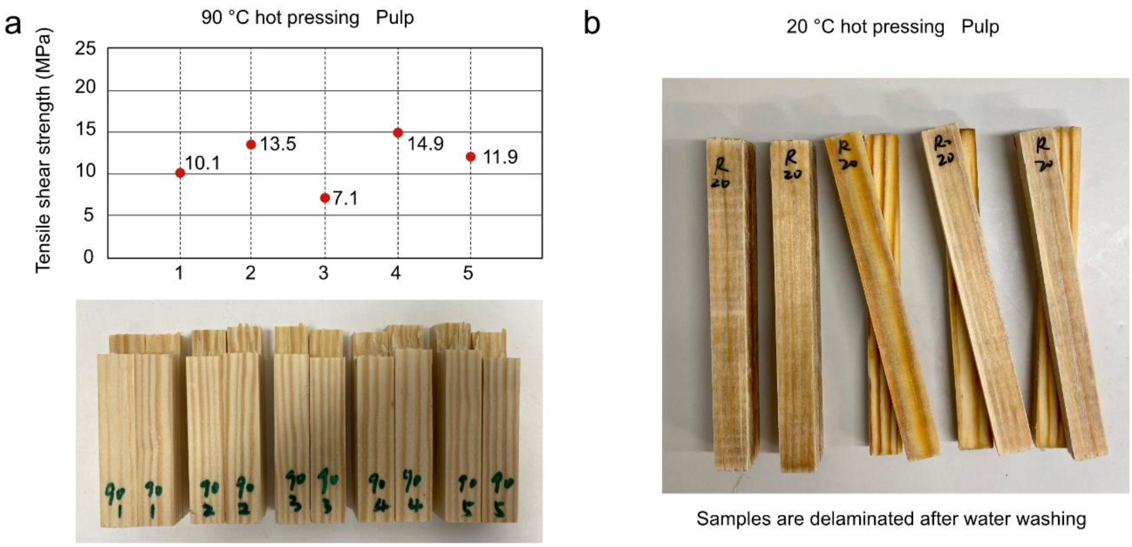

Supplementary Fig. 21. Effect of bonding temperature. The shear strength and photos of fractured wood samples bonded with 5 wt.% pulp-IL solution and hot-pressed at different temperatures for 30 min. a) wood sample hot-pressed at 90 °C with 1.5 MPa pressure. b) wood sample hot-pressed at 20 °C with 1.5 MPa pressure. c) wood sample hot-pressed at 140 °C with 1 MPa pressure. d) wood sample hot-pressed at 140 °C with 0.1 MPa pressure.

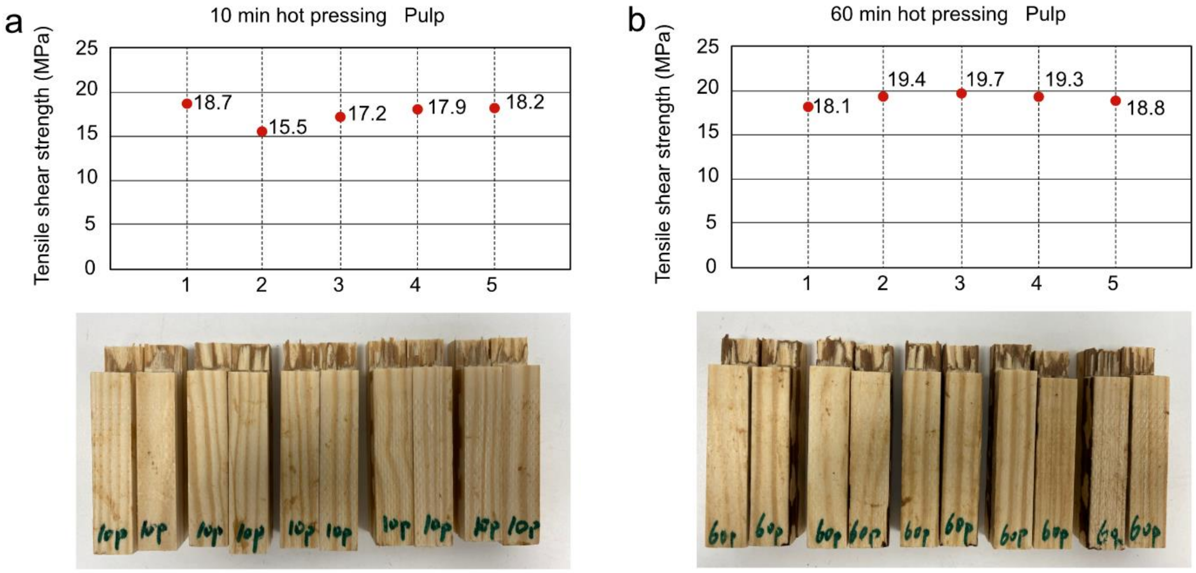

Supplementary Fig. 22. Effect of hot-pressing duration. The bonding strengths and the images of fractured wood samples bonded with 5 wt.% pulp-IL solution and hot-pressed for different durations at 140 °C with 1.5 MPa pressure. a) wood sample hot-pressed for 10 min. b) wood sample hot-pressed for 60 min.

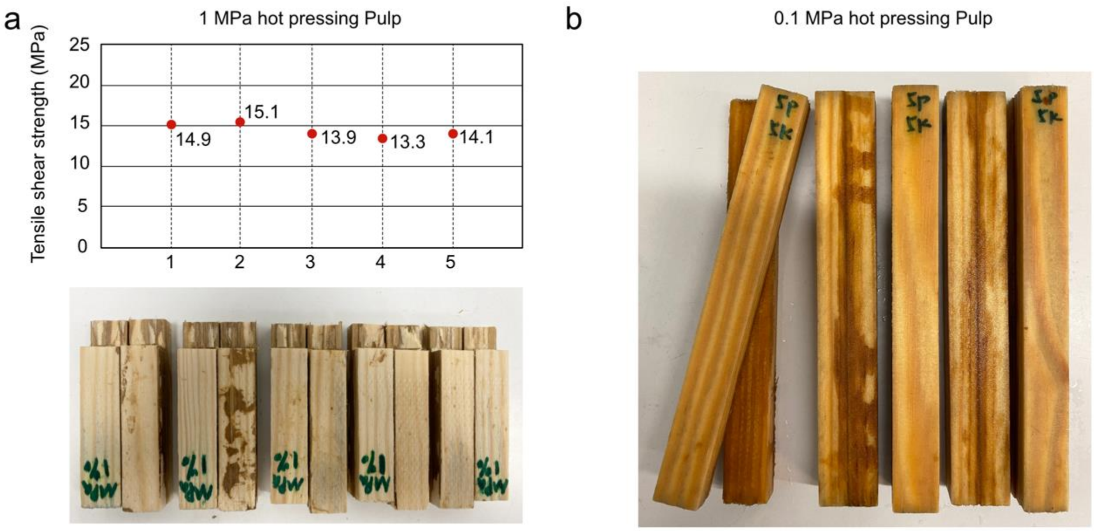

Supplementary Fig. 23. Effect of hot-pressing pressure. The bonding strengths and the images of fractured wood samples bonded with 5 wt.% pulp-IL solution and hot-pressed at different pressures for 30 min. a) wood sample hot-pressed at 140 °C with 1 MPa pressure. b) wood sample hot-pressed at 140 °C with 0.1 MPa pressure.

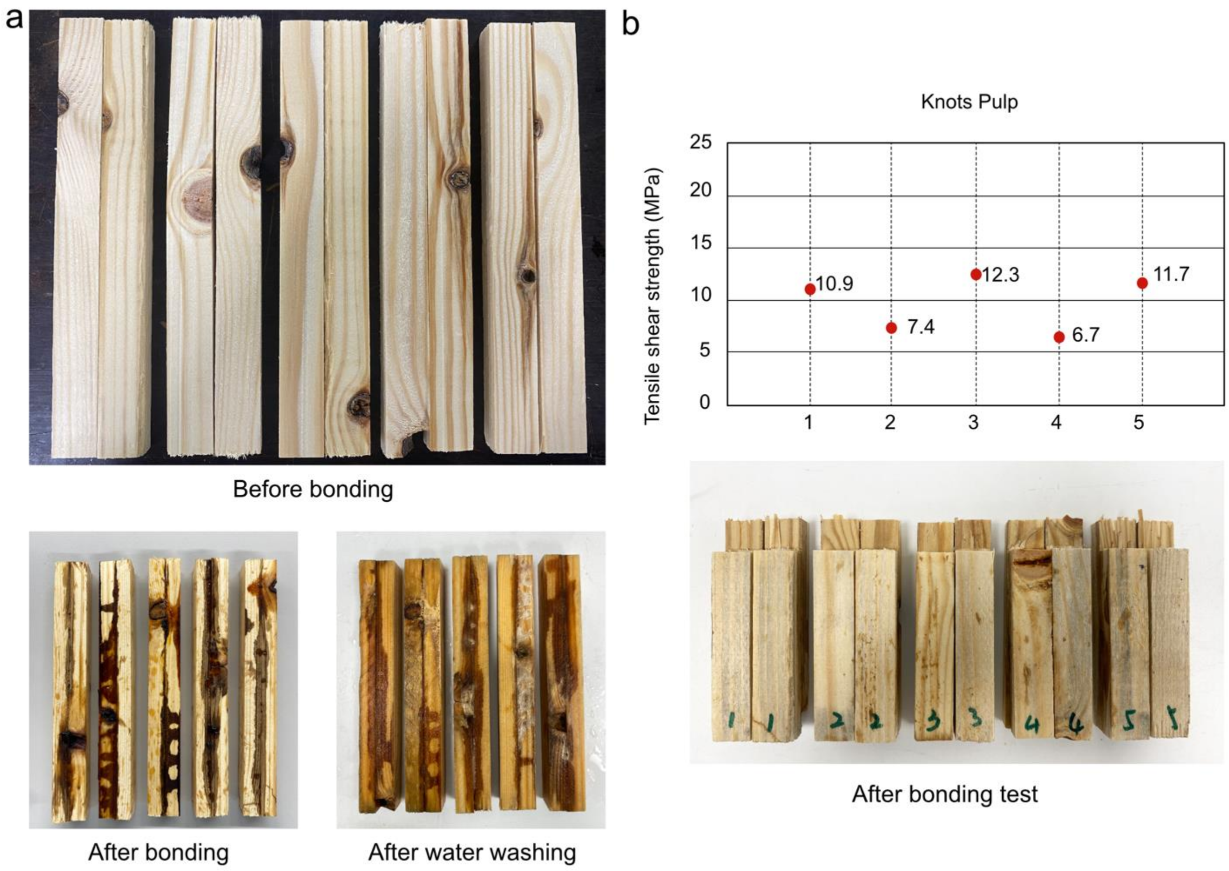

Supplementary Fig. 24. Photos of wood substrates containing knots and the corresponding bonding strength of samples. a) images of wood substrates containing knots before and after bonding with 5 wt.% pulp-IL solution and hot-pressed at 140 °C with 1.5 MPa pressure for 30 min, and subsequent regeneration in water. b) the bonding strength and the image of fractured wood samples containing knots.

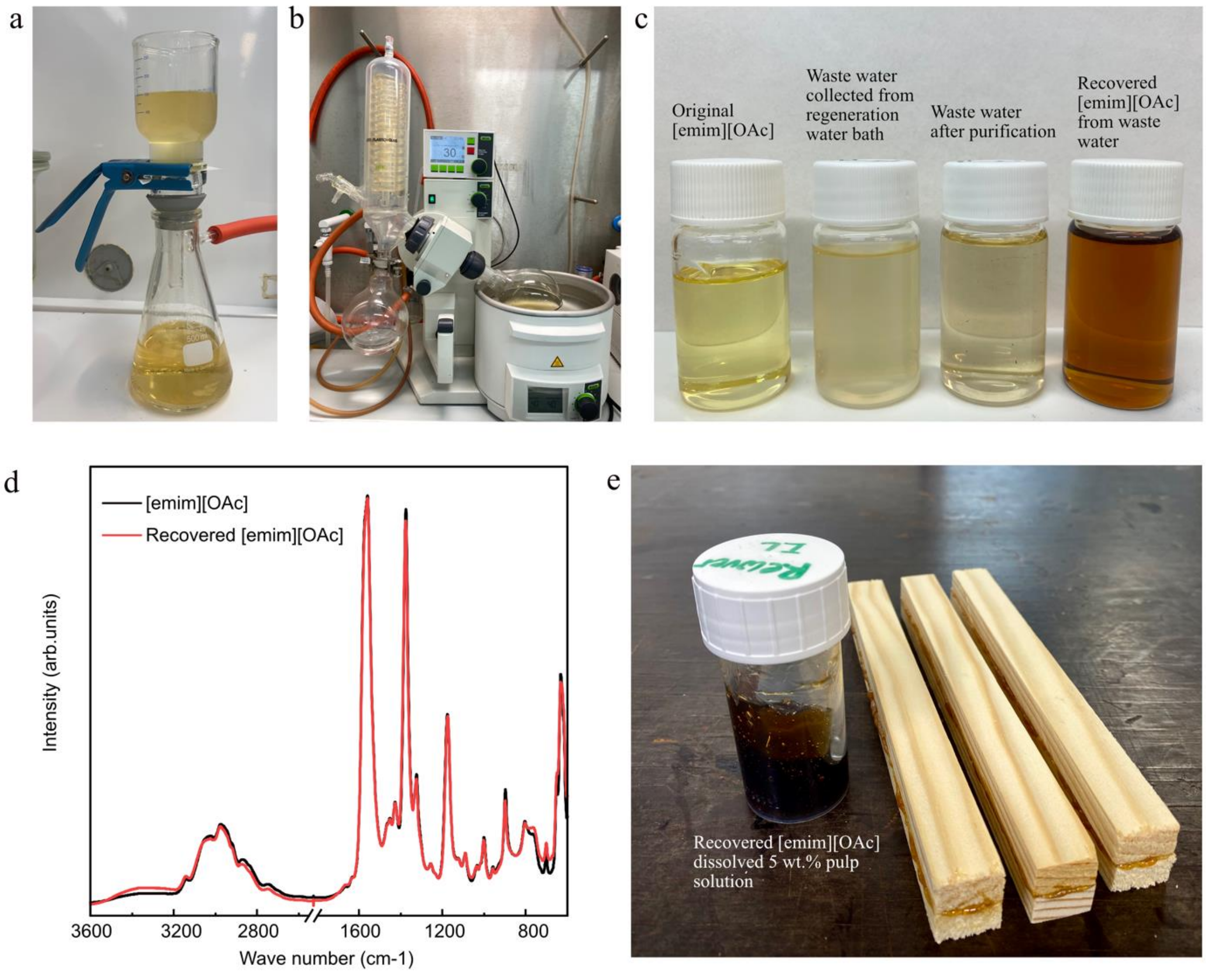

Supplementary Fig. 25. Purification, recovery, and re-use of IL [emim][OAc]. a) purification setup for removing insoluble fraction from [emim][OAc]-containing wastewater. b) rotary evaporation process for removing water from the purified [emim][OAc]/water mixture. c) photo showing the original [emim][OAc], collected wastewater, purified wastewater, and recovered [emim][OAc]. d) FTIR spectra comparing the original [emim][OAc] and recovered [emim][OAc]. e) photo of a 5 wt.% pulp solution dissolved in the recovered [emim][OAc] and the resulting wood assemblies.

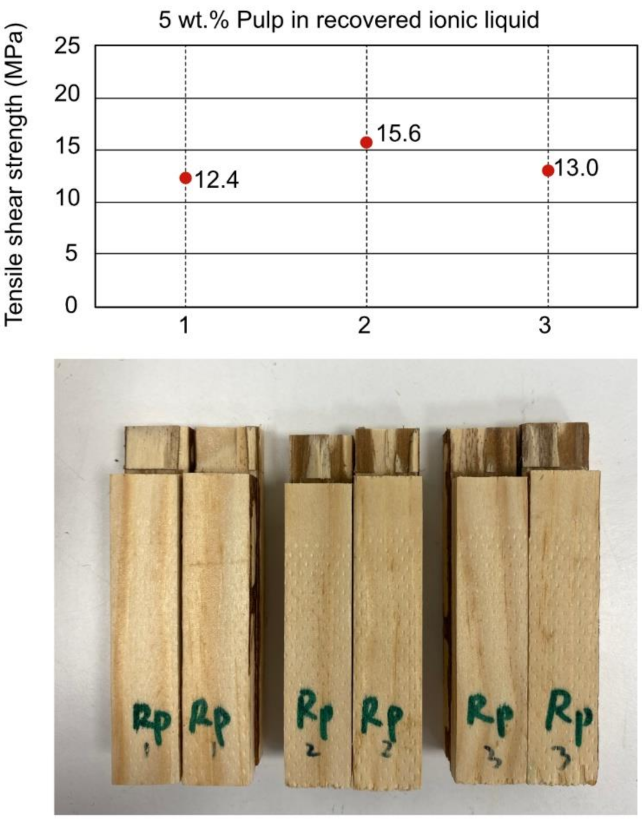

Supplementary Fig. 26. Shear strength of wood bonded with 5 wt.% pulp solution prepared using recovered [emim][OAc], along with corresponding photo of the fractured specimens.

Supplementary Table 1. The compound cell wall thickness was measured before swelling, after swelling in the pulp-IL solution at 60 °C, and after water regeneration.

*Cell wall thickness (μm)						
Cell wall label	0 min 20 °C	60 min 20 °C	10 min 60 °C	20 min 60 °C	30 min 60 °C	After rinsing
Earlywood	6.1 [0.8]	7.2 [1.0]	12.6 [1.6]	13.5 [2.0]	14.4 [2.4]	9.7 [1.5]
Latewood	10.6 [2.0]	11.6 [2.5]	18.2 [1.4]	19.6[1.1]	20.9[0.4]	14.7 [1.7]

* The mean values, along with standard deviations (in bracket), of the compound cell wall thickness were determined from three samples. For each sample, measurements were taken at five points on both earlywood and latewood, respectively.

Supplementary Table 2. The engineering shear strength and stiffness of the sample bonding lines with respective standard deviation in bracket.

Sample	Dry shear strength (MPa)	Wet shear strength (MPa)	Strain to failure (%)	Shear Stiffness (MPa)
Solid Wood	12.2 [1.0]	-	3.8 [0.8]	392.3 [39.9]
MCC (no regeneration)	11.9 [1.2]	-	3.4 [0.6]	399.7 [25.5]
MCC	15.7 [3.0]	-	4.2 [0.7]	526.7 [57.9]
MFC	17.2 [1.0]	-	4.7 [0.6]	508.0 [39.1]
Pulp	19.6 [1.3]	-	5.1 [1.0]	495.6 [58.6]
Pulp after boiling	17.8 [0.7]	-	-	-
Plywood (pulp IL)	4.9 [0.7]	1.7 [0.7]	-	-
Plywood (phenol formaldehye)	2.2 [0.4]	-	-	-

Supplementary Table 3. Bonding method, wood species, wood density, and shear strength of bonded wood assemblies reported in literatures.

Bonding method	Wood	Wood density (kg/m^3)	Shear strength (MPa)	Ref.
Dialdehyde cellulose	Beech	660 [20]	9.53 [0.07]	3
	Norway spruce	380 [20]	5.75 [0.14]	
Resorcinol resin (RF)	China fir	407	7.5 [0.4]	4
	Japanese cedar	452	8.2 [0.2]	
	Taiwania	420	7.5 [0.1]	
	Douglas fir	503	9.3[0.2]	
	Southern pine	530	8.0 [0.3]	
Melamine urea formaldehyde/CMC	Douglas fir	503	10.1	5
Phenol resorcinol formaldehyde	Douglas fir	503	8.4	
Melamine urea formaldehyde	Eucalyptus	564	5.51-6.81	6
Polyvinyl acetate (PVAc)	Beech	680	8.79 [3.44]	7
Phenol resorcinol formaldehyde	Beech	680	11.17 [2.01]	
Polyurethane (PU)	Eucalyptus	524	11.2	8
Phenol resorcinol formaldehyde	*Acacia mangium*	637 [92]	10.5 [1.8]	9
Polyurethane	*Acacia mangium*	637 [92]	8.7 [4.8]	
Polyvinyl acetate (PVAc)	Calabrian pine	560	7.54-7.91	10
Polyurethane (PU)	Calabrian pine	560	6.15-6.97	
Polyurethane (PU)	Scots pine	473[30]	12.1[1.5]	11
Polyvinyl acetate (PVAc)	Scots pine	473[30]	10.2[1.5]	
Melamine urea formaldehyde	Scots pine	473[30]	9.7[1.5]	
Phenol resorcinol formaldehyde	Scots pine	473[30]	10.7[0.9]	

Mechanical welding	Spruce	410	2.1	12
	Birch	568	7.9	
Mechanical welding	Birch	561	6.0	13
	Beech	657	8.1	
Mechanical welding	Pine	480	4.1	14
Mechanical welding	Spruce	380	2.0	15
	Beech	680	7.6	
Solid wood	Alder, red	410	7.4	16
	Basswood	370	6.8	
	Beech	640	13.9	
	Birch, yellow	620	14.0	
	Pine	550	13.0	
	Maple	480	11.9	
	Maple	630	16.1	
	Oak, red	610	13.2	
	Pine	680	14.4	
	Yellow-poplar	420	8.2	
	Cedar	320	5.5	
	Pine	420	11.0	
	Douglas-fir	480	7.8	
	Spruce, Sitka	400	7.9	
	Spruce	440	10.3	
	Spruce, red	400	8.9	

Standard deviation in bracket.

Supplementary Reference

1 Dahl, K. B. & Malo, K. A. Linear shear properties of spruce softwood. *Wood Science and Technology* **43**, 499-525 (2009). https://doi.org/10.1007/s00226-009-0246-5

2 Yoneda, Y., Hettegger, H., Böhmdorfer, S., Potthast, A. & Rosenau, T. Cellulose acylation in aged 1-ethyl-3-methyl-imidazolium carboxylate ionic liquids upon fiber spinning. *Cellulose* (2025). https://doi.org/10.1007/s10570-025-06583-y

3 Zhang, H., Liu, P. W., Musa, S. M., Mai, C. & Zhang, K. Dialdehyde Cellulose as a Bio-Based Robust Adhesive for Wood Bonding. *Acs Sustain Chem Eng* **7**, 10452-10459 (2019). https://doi.org/10.1021/acssuschemeng.9b00801

4 Yang, T.-H., Wang, S.-Y., Tsai, M.-J., Lin, C.-Y. & Chuang, Y.-J. Effect of fire exposure on the mechanical properties of glued laminated timber. *Mater. Des.* **30**, 698-703 (2009). https://doi.org/10.1016/j.matdes.2008.05.022

5 Zhou, J. *et al.* Bonding performance of melamine-urea-formaldehyde and phenol-resorcinol-formaldehyde adhesives in interior grade glulam. *J. Adhes. Sci. Technol.* **31**, 2630-2639 (2017). https://doi.org/10.1080/01694243.2017.1313185

6 Alade, A. A., Naghizadeh, Z., Wessels, C. B., Stolze, H. & Militz, H. Adhesion performance of melamine-urea-formaldehyde joints of copper azole-treated Eucalyptus grandis at varied bonding process conditions. *Constr. Build. Mater.* **314** (2022). https://doi.org/10.1016/j.conbuildmat.2021.125682

7 Adamopoulos, S., Bastani, A., Gascón-Garrido, P., Militz, H. & Mai, C. Adhesive bonding of beech wood modified with a phenol formaldehyde compound. *Eur. J. Wood Prod.* **70**, 897-901 (2012). https://doi.org/10.1007/s00107-012-0620-0

8 Pröller, M. *An investigation into the edge gluing of green Eucalyptus grandis lumber using an one-component polyurethane adhesive*, Stellenbosch University, (2017).

9 Mohd Yusof, N. *et al.* Bond integrity of cross laminated timber from Acacia mangium wood as affected by adhesive types, pressing pressures and loading direction. *Int. J. Adhes. Adhes.* **94**, 24-28 (2019). https://doi.org/10.1016/j.ijadhadh.2019.05.010

10 Burdurlu, E., Kiliç, Y., Elibol, G. C. & Kiliç, M. The shear strength of Calabrian Pine (Ten.) bonded with polyurethane and polyvinyl acetate adhesives. *J. Appl. Polym. Sci.* **99**, 3050-3061 (2006). https://doi.org/10.1002/app.22905

11 Wang, X. D. *et al.* Shear Strength of Scots Pine Wood and Glued Joints in a Cold Climate. *Bioresources* **11**, 944-956 (2016). https://doi.org/10.15376/biores.11.1.944-956

12 Stamm, B., Natterer, J. & Navi, P. Joining wood by friction welding. *J. Adhes. Sci. Technol.* **19**, 1129-1139 (2005). https://doi.org/10.1007/s00107-005-0007-6

13 Boonstra, M. *et al.* Vibration welding of heat-treated wood. *J. Adhes. Sci. Technol.* **20**, 359-369 (2006). https://doi.org/10.1163/156856106776381758

14 Mansouri, H. R. *et al.* Causes for the Improved Water Resistance in Pine Wood Linear Welded Joints. *J. Adhes. Sci. Technol.* **25**, 1987-1995 (2012). https://doi.org/10.1163/016942410x544794

15 Gfeller, B. *et al.* Wood bonding by mechanically‐induced in situ welding of polymeric structural wood constituents. *J. Appl. Polym. Sci.* **92**, 243-251 (2004). https://doi.org/10.1002/app.13648

16 Ross, R. *Wood handbook: Wood as an engineering material*. (U.S. Department of Agriculture, Forest Service, Forest Products Laboratory, 2021).
