# Supplementary material for: Multiscale interface engineering enables strong and water resistant wood bonding
Source: Nat Commun. 2025 Aug 25;16:7902. doi: 10.1038/s41467-025-63239-y (PMC12378965; doi:10.1038/s41467-025-63239-y)
Supplement: Supplementary file 2 — Description of Additional Supplementary Files [file 41467_2025_63239_MOESM2_ESM.pdf]

Description of Additional Supplementary Files

Supplementary Movie 1:

Tensile shear test of bonded wood assembly at a speed of 5 mm min-1

Supplementary Movie 2:

Delamination test of pulp-bonded wood assembly. Total test duration: 6 hours 18 mins. (Movie shortened).

Supplementary Movie 3:

Regeneration of ionic liquid–dissolved cellulose in bonded wood assembly using hot steam.
